# Protein interactions in human genetic diseases

**DOI:** 10.1186/gb-2008-9-1-r9

**Published:** 2008-01-16

**Authors:** Benjamin Schuster-Böckler, Alex Bateman

**Affiliations:** 1 Wellcome Trust Sanger Institute, Wellcome Trust Genome Campus, Hinxton, CB10 1SA, UK

## Abstract

We present a novel method that combines protein structure information with protein interaction data to identify residues that form part of an interaction interface. Our prediction method can retrieve interaction hotspots with an accuracy of 60% (at a 20% false positive rate). The method was applied to all mutations in the Online Mendelian Inheritance in Man (OMIM) database, predicting 1,428 mutations to be related to an interaction defect. Combining predicted and hand-curated sets, we discuss how mutations affect protein interactions in general.

## Abstract 2

A method is presented to identify residues that form part of an interaction interface, leading to the prediction that 1,428 OMIM mutations are related to an interaction defect.

## Background

Interactomics, the study of physical interactions between biological molecules, is establishing itself as a complementary approach to decode biological function. The growing flood of molecular interaction data has been compared to the development of genome sequencing in the past decade [1]. More than 20,000 human protein interactions have been deposited in protein interaction databases [2] and many more can be inferred from other model organisms. Despite the fact that these interactions are assumed to constitute only a fraction of the full protein interaction network in a human cell [3], the data can already provide valuable information [4,5].

A wealth of investigations have been undertaken to deepen our understanding of hereditary diseases. As a result of that, databases such as the Online Mendelian Inheritance in Man (OMIM) [6] and UniProt [7] together contain almost 30,000 experimentally verified mutations. Nevertheless, the exact mechanisms by which mutations alter a protein's function are in many cases poorly understood. Most of the known disease-related mutations are non-synonymous single nucleotide polymorphisms in the coding regions of a gene (nsSNPs) [8], although stop and nonsense mutations play a role in a number of hereditary diseases, too [9]. Recent studies also stress the importance of changes in splicing and post-translational modification as causes of disease [10]. It has been suggested that up to 80% of disease-associated nsSNPs destabilize the protein through steric or electrostatic effects [8,11]. Ferrer-Costa *et al. *[12] compared disease-associated and neutral nsSNPs in 73 proteins and estimated that 10% of disease-associated nsSNPs may affect the quaternary structure of the protein, thereby changing protein interactions.

In this study, we focus on those diseases that are caused by mutations in protein interaction interfaces. In recent years, some interaction-related diseases, such as Alzheimer's and Creutzfeldt-Jacob disease, have received much attention [4,13,14]. These conditions feature an induced aggregation of proteins, often called amyloidosis. Furthermore, diseases can also be caused by the disruption of protein binding. A typical example is Charcot-Marie-Tooth disease, which can be triggered by the loss of interaction between myelin protein zero monomers that link adjacent membranes of the myelin sheath [15]. To our knowledge, neither type of interaction-related mutations has yet been studied in a systematic way.

We describe a method that combines protein structure with experimental protein interaction data in order to computationally identify residues that form part of a binding interface. We apply this algorithm to mutations from OMIM and UniProt, identifying 1,428 mutations that are likely to affect protein interactions. Subsequently, we collected numerous topical reports of changes in protein interaction that result in disease. We present a list of 119 interaction-related mutations causing 65 different diseases that was derived manually from the scientific literature. On the basis of these sets we discuss general properties of interaction-related mutations.

## Results and discussion

### Prediction algorithm

In order to identify residues in a protein that are involved in a protein interaction, we devised a method that combines structural and experimental information. Using the *i*Pfam [16] database of known interacting domains, we first select domain regions on all target proteins that have a homologous structure including interaction partners in the PDB [17] (see Materials and methods). We then select positions that form residue-to-residue contacts between distinct polypeptide chains in these structural templates and record the corresponding positions in the target proteins as potentially interacting residues.

We needed to choose a scoring function that discriminates between residues that are really involved and crucial for an interaction and those that are not. For this purpose, we tested the effect of two different variables on prediction accuracy.

#### Percent sequence identity with structural template

**Figure 1:**
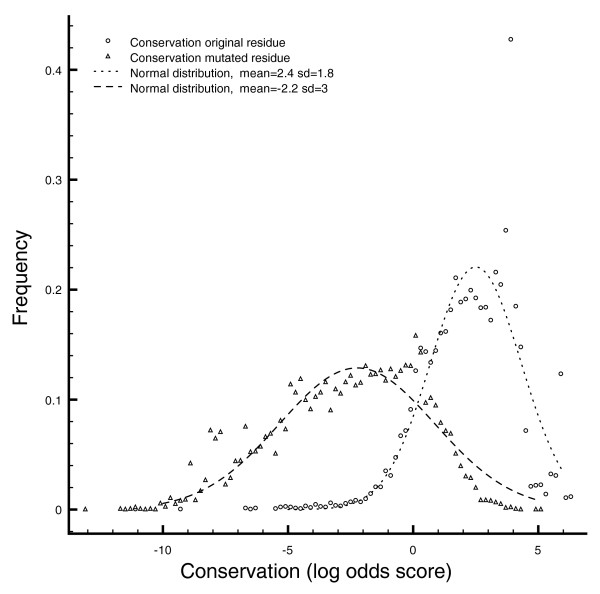
Conservation difference between wild-type and mutated residues. Histogram of conservation of wild-type and mutated residues. Triangles denote the residue-conservation frequency of all residues in disease protein regions that map to an *i*Pfam domain. Circles show the conservation of the pathogenic alleles (see Materials and methods). Trendlines are added to delineate normal distributions.

There is a well known correlation between sequence similarity and structural similarity [18], which also extends to interacting domains [19]. An interaction is more likely to be conserved and to display similar topology when sequence similarity is high. Although we find that percentage identity by itself is not a good predictor of the importance of a residue for an interaction, it can improve the prediction accuracy slightly when combined with another threshold (Figure 1).

#### Conservation of mutated residues

**Figure 2:**
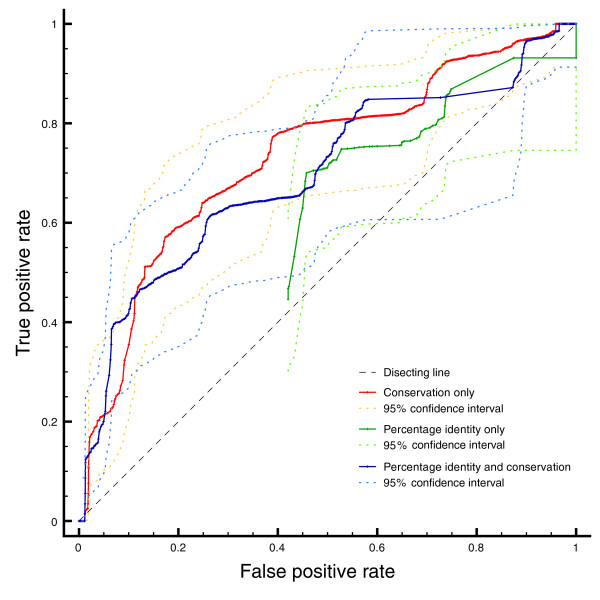
ROC curves calculated on a set of alanine scanning experiments. The red line represents the performance of our algorithm when changing only the conservation threshold, applying no percentage identity cutoff. The green line shows the performance using only percentage identity as a threshold. The blue line reflects performance using conservation as threshold, but applying a 30% sequence identity filter. Confidence intervals where calculated using the Statistics::ROC Perl module [59].

For all identified interaction-related mutations, we calculated a conservation score (see Materials and methods). This score reflects the frequency with which an amino acid occurs at a given position in a protein family, relative to a universal background distribution. If we look at the frequency of conservation scores over all wild-type compared to all mutated alleles (Figure 1), we find that the scores for both wild-type as well as mutated alleles seem to follow a normal distribution. However, the latter exhibit markedly smaller average conservation scores (2.4 versus -2.2; Figure 2). Thus, a residue that is found in the wild type of a protein will generally be more conserved than the residue found in the mutated version [20]. We therefore tested whether conservation could be used as an indicator of the functional importance of a residue, even for surface exposed residues like the ones under investigation here.

### Prediction accuracy

To estimate the accuracy of our prediction approach, we used the ASEdb database of alanine scanning energetics experiments in protein binding [21] as a 'gold-standard' test set (see Materials and methods). In such an alanine scan, residues in the binding interface of a protein are mutated to alanine by site-directed mutagenesis [22]. The difference in binding free energy (ΔΔ*G*) between wild-type (Δ*G*_0_) and mutated (Δ*G*_*A*_) protein describes the contribution of a particular residue at position *i *to the total binding free energy:

ΔΔ*G*_*i *_= Δ*G*_*O *_- *G*_*A*,*i*_

We assessed how well our method could predict residues with a large change in Δ*G *upon mutation. Randles *et al. *[23] showed that for two model proteins, ΔΔ*G *was correlated with the severity of disease. They show that even changes <2 kcal/mol could cause disruption of protein binding. Here, we defined a residue as correctly identified (true positive) if ΔΔ*G *> 2.5. This threshold is also used in another recent publication [24]. Residues below this threshold were considered neutral (false positive). This criterion might in itself cause some 'false-negatives', that is, some residues might be crucial for the function of the protein despite a measured ΔΔ*G *< 2.5, but we considered a conservative threshold to be preferable.

Figure 1 shows the receiver operator characteristic (ROC) curve [25], a plot of the frequency of true positive over the frequency of false positive predictions for a given algorithm. From left to right, points mark decreasing score thresholds, until no thresholds are applied any more and both true positive as well as false positive rates reach 100% in the upper right corner.

The green and red lines represent the performance of our algorithm using either percentage sequence identity (green) or residue conservation (red) to score the predictions. With both scoring methods, our method retrieves more true positives than would be expected by chance. The conservation threshold, however, is far superior in distinguishing true from false positives. At a false positive rate of ≈20%, we can achieve a true positive rate of almost 60%. These benchmark results underline that we are able to identify interaction disruptive mutations with reasonable confidence. The real accuracy could be even higher than measured here, considering the conservative ΔΔ*G *cutoff we chose to define a true positive residue.

We also tested a combination of the two measures, represented by a blue line in Figure 1. In this case, the residue conservation threshold was combined with a fixed 30% sequence identity cutoff. The performance improves slightly in the low false-positive region, yielding a true positive rate of 40% at a false positive rate of only 7%. In accordance with this benchmark, we decided on a residue conservation threshold of >2 in combination with a 30% sequence identity cutoff for all subsequent analyses. In order to make our algorithm generally applicable, two more filters were applied: target proteins had to have a homologous sequence (BLAST e-value of less than 10^-6^) in one of four major repositories for protein interaction information (IntAct [26], BioGRID [27], MPact [28] or HPRD [29]). Subsequently, target proteins were excluded if no homologous experimental interaction involved both interacting *i*Pfam domains that were seen in the structural template.

### Application to disease mutations

We applied the prediction algorithm as described above to all single-residue disease mutations extracted from OMIM and UniProt (see Materials and methods). In the case of disease mutations, the disruptive nature of a residue mutation is already known. It is unclear, however, whether an interaction is in fact taking place and is likely to be mediated by the domain in question. As described above, mutations were reported, therefore, only if the disease associated protein has a close homolog that has been proven experimentally to interact with a protein that contains the same binding partner domain as seen in the PDB structure the interaction was modeled from (the 'structural template'). For example, [OMIM:+264900.0011] is a Ser576Arg mutation of the human coagulation factor IX (PTA). The residue is part of a trypsin domain and seen to interact with Ecotin. However, the interaction between PTA and Ecotin is not yet recorded in any interaction database; therefore, the mutation cannot be included in our predictions.

**Figure 3:**
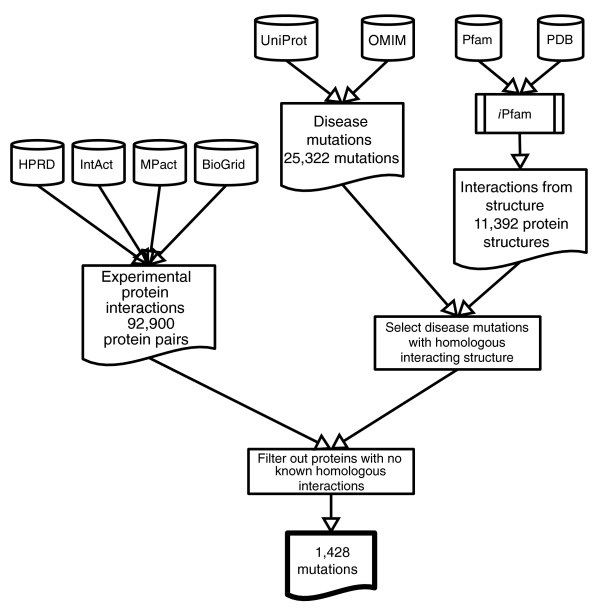
Data integration steps for interacting residue prediction. Schematic outline of data integration for the prediction of interacting residues. Mutations from OMIM and UniProt for which a residue in a homologous structure is involved in an interaction are selected. This set is restricted further by searching for homologous proteins with known interactions, taken from a range of protein interaction databases. We require that the the homologous interacting proteins contain the same pair of Pfam domains that was observed in the structural template. This results in a set of 1,428 interaction related mutations.

Using these criteria, 1,428 mutations from 264 proteins were predicted to be interaction-related (Figure 3). The full list is available in Additional data file 1. In total, we collected 25,322 mutations from OMIM and UniProt. This means that approximately 4% of all mutations could be linked to a protein interaction.

Amongst these mutations, 454 mapped to a structure that exhibits an interaction between different proteins (hetero-interaction), while 1,094 mutations mapped to a structure with an interaction between two identical proteins (homo-interaction). This means that 120 mutations are found in structures of both homo- and hetero-interactions. The large proportion of homo-interactions can be explained by the overrepresentation of homo-interactions in the structural templates set: 70% of all distinct protein pairs in *i*Pfam are homo-interactions, which is in accordance with recent findings that homo-interactions are more common than hetero-interactions [30].

### Properties of mutations in interaction interfaces

#### Curated set of interaction-related mutations

In addition to the automatically derived data, we collected 119 mutations in 65 distinct diseases from the scientific literature for which there is evidence that they change the interactions of the protein they occur in (see Materials and methods). We call this the 'curated set' of interaction-related mutations (Additional data file 2). To our knowledge, it represents the biggest collection of high confidence interaction-related mutations to date.

Below, we explore differences between interaction-related mutations and non-interaction-related mutations. We focus on the mechanism of the mutation, the mode of inheritance and residue composition. For most of the 1,428 mutations from the automatically generated set, no information about their mode of inheritance or functional mechanism was instantly available. To allow a comparison with the manually curated set, we sampled 100 mutations randomly and conducted a manual search of the literature in order to annotate their properties.

#### Classification according to function

We suggest a classification that groups mutations according to their effects into loss of function (LOF) and gain of function (GOF). Below this broad distinction, the GOF mutations can be further divided into two groups: pathological aggregation and aberrant recognition. Similarly, LOF mutations can be split into one class that disrupts obligate interactions between protein subunits and another class that interferes with transient interactions.

From the curated set of interaction-related mutations, 95 mutations result in LOF, 17 in GOF, 4 mutations were reported to change the interaction preference of the protein and 3 could not be determined. The class of GOF mutations that result in protein aggregation contains 12 cases, comprising amyloid diseases like Alzheimer's or Creutzfeldt-Jacob, but also, for example, sickle cell anemia [OMIM:+141900.0243]. Five cases result in aberrant recognition; for example, a Gly233Val mutation in glycoprotein Ib that leads to von Willebrand disease [OMIM:*606672.0003] by increasing the affinity for von Willebrand factor.

Amongst the LOF mutations, 61 affect transient interactions and 34 affect obligate interactions. The latter usually render proteins dysfunctional, for example, in the case of lipoamide dehydrogenase deficiency caused by impaired dimerization [31]. LOF mutations in transient interactions cause changes in localization or transmission of information, exemplified by a mutation in the *BRCA2 *gene that predisposes women to early onset breast cancer: a Tyr42Cys mutation in *BRCA2 *inhibits the interaction of BRCA2 with replication protein A, a protein essential for DNA repair, replication and recombination [32]. Lack of this interaction inhibits the recruitment of double stranded break repair proteins and eventually leads to an accumulation of carcinogenic DNA changes.

#### Mode of inheritance

We investigated the mode of inheritance for all mutations in the curated set, if information was available in the literature. All GOF mutations showed dominant inheritance (the two hemoglobin mutations exhibit incomplete dominance). Out of 61 LOF mutations for which inheritance information was available, 24 were autosomal dominant and 37 were recessive. Jimenez-Sanchez *et al. *[33] studied the mode of inheritance of human disease genes. According to them, mutations in enzymes are predominantly recessive, while mutations in receptors, transcription factors and structural proteins are often dominant. Overall, they find a ratio of 188:335 of dominant to recessive diseases. In our data set, the ratio of dominant to recessive mutations is 41:37 (31:29 in terms of diseases). This enrichment for dominant mutations is statistically significant, as determined by a two-sided test for equality of proportions (*P-*value < 0.014). The increase was seen across Gene Ontology functional categories, in enzymes as well as regulators and signaling proteins (data not shown). In the 100 randomly chosen mutations from the predicted set, we found a ratio of dominant to recessive mutations of 38:41, which is very similar to the ratio observed in the curated set (two-sided test for equality of proportions; *P-*value > 0.68; hypothesis of difference in proportions rejected).

In GOF mutations, dominant inheritance is not surprising, but the high proportion (39%) of dominant LOF mutations is noteworthy. Dominant inheritance in LOF mutations can be explained by either haploinsufficency or dominant negative effects [34]. In yeast, dosage sensitivity of members of protein complexes has been shown [35]. According to what Papp *et al. *call the 'balance hypothesis', stoichiometric imbalances have negative effects on the function of protein complexes. Dominance would thus be a result simply of a lack of functional protein subunits.

Dominant negative effects as a result of interallelic complementation could be an alternative explanation for the observed enrichment of dominant mutations. For example, mutations of phenylalanine hydroxylase can lead to phenylketonuria [36] by inhibiting necessary conformational changes between monomers. In such cases where the protein function relies on the dynamic interactions between subunits, a mutation in one of the binding interfaces can actively inhibit the function of the other bound members of the complex. Detailed experimental analysis of dominant LOF mutations could reveal the relative importance of dominant negative effects compared to haploinsufficency due to stoichiometric imbalances.

#### Residue frequency

The residue frequency of the predicted interaction-related mutations was compared to the frequencies of residues over all mutation in OMIM and UniProt [37]. We find that the frequency distribution of wild-type residues in interaction-related mutations is mostly similar to the overall mutational spectrum, with the exceptions of a significant enrichment in glycine and, to a lesser extent, a higher frequency of tryptophan and glutamine and a reduced frequency of alanine, serine and valine (figure in Additional data file 3). The enrichment in glycine can not be readily explained by the composition of residues on the protein surface or in interaction interfaces [38,39] but might be due to the disruptive nature of the residues glycine is most likely to mutate to, namely arginine, serine and aspartate [37].

### Examples of putative interaction-related mutations

In the following section we describe three diseases identified by our method that appear likely to be related to changes in protein interaction.

#### Griscelli syndrome, type 2 [OMIM:#607624]

**Figure 4:**
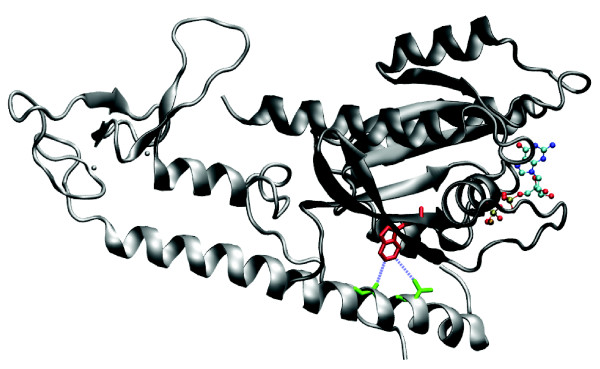
Structure of *Rattus norvegicus *Ras-related protein Rab-3A [PDB:1ZBD]. The small G protein Rab3A with bound GTP interacting with the effector domain of rabphilin-3A. The residue corresponding to the mutated Trp73 from human RAB27A is highlighted in red, while the two residues in contact with it are coloured green.

Griscelli syndrome is a disease that features abnormal skin and hair pigmentation as well as, in some cases, immunodeficiency due to a lack of gammaglobulin and insufficient lymphocyte stimulation. Without bone marrow transplantation, the disease is usually fatal within the first years of life [40]. The type 2 form of Griscelli syndrome usually maps to the Rab-27A gene [41]. The RAS domain of Rab-27A shares 46.8% sequence identity with the same domain in Ras-related protein Rab-3A from *Rattus norvegicus*. The crystal structure of Rab-3A interacting with Rabphilin-3A was solved by Ostermeier and Brunger [42] (PDB:1ZBD; Figure 4). We found that a Trp73Gly mutation in Rab-27A affects a residue that is both highly conserved (scores of 5.62 for tryptophan and -1.84 for glycine) and in the center of the interaction interface. There is strong evidence that Rab-27A interacts with Myophillin [43]. For these reasons the Trp73Gly mutation seems likely to affect vesicle transport by reducing affinity of Rab-27A to Myophilin.

#### Adrenocorticotropin hormone deficiency [OMIM:#201400]

**Figure 5:**
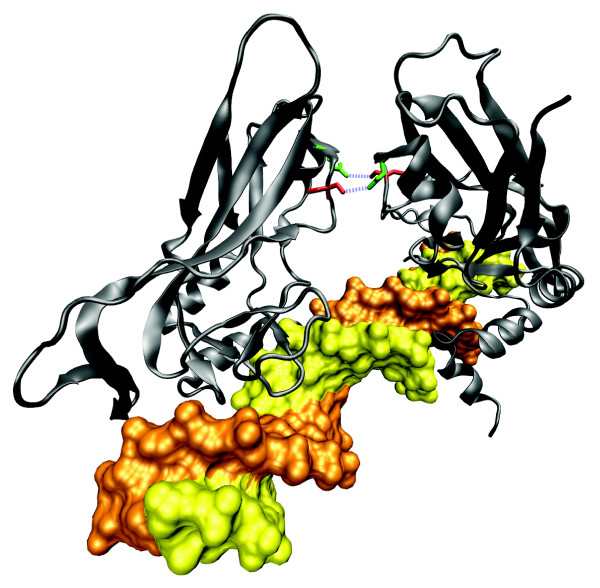
Structure of *X. laevis *Brachyury protein [PDB:1XBR]. The crystal structure of a T-domain from *X. laevis *bound to DNA. The residues highlighted in red are the mutated Ser128, with green residues representing the contact residues in the partner protein. Blue dashed lines show residue contacts.

Adrenocorticotropin hormone deficiency is characterized by a marked decrease of the pituitary hormone adrenocorticotropin and other steroids. Its symptoms include, amongst others, weight loss, anorexia and low blood pressure. Lamolet *et al. *[44] identified a Ser128Phe mutation in the T-box transcription factor TBX19 that leads to a dominant LOF phenotype [UniProt:O60806, VAR_018387]. The crystal structure of the homologous T-Box domain from the *Xenopus laevis *Brachyury transcription factor [45] (81% sequence identity to the human TBX19 protein; [PDB:1XBR]) shows that this particular residue is at the core of the dimerization interface (Figure 5). The mutation substitutes a small polar with a large aromatic side-chain. Accordingly, the residue features strong conservation, while phenylalanine is very rare at this position (scores of 3.31 and -1.78 for serine and phenylalanine, respectively). Pulichino *et al. *[46] report that the Ser128Phe mutation shows virtually no DNA binding affinity. We predict that this loss of affinity is due to a drop in binding free energy between monomer and DNA, as compared to the dimer.

#### Baller-Gerold syndrome [OMIM:#218600]

Baller-Gerold syndrome is a rare congenital disease characterized by distinctive malformations of the skull and facial area as well as bones of the forearms and hands. The disease phenotypically overlaps with other disorders like Rothmund-Thomson syndrome or Saethre-Chotzen syndrome. Seto *et al. *[47] reported a case of Baller-Gerold syndrome that also included features of Saethre-Chotzen syndrome. They identified an isoleucine to valine substitution at position 156 of the H-Twist protein as the causative mutation. Experimental studies using yeast-two-hybrid assays have reported the loss of H-Twist/E12 dimerization ability as a possible cause of Saethre-Chotzen syndrome [48].

**Figure 6:**
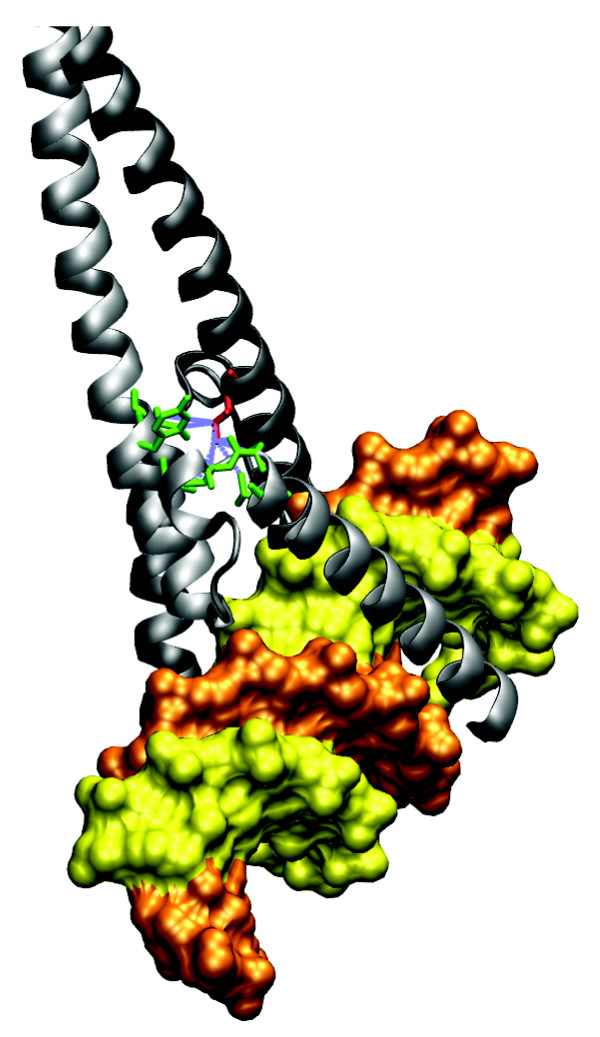
Structure of the Myc/Max transcription factor complex binding DNA [PDB:1NKP]. Both Myc-c and Max form a basic helix-loop-helix motif. They dimerize mainly through their extended helix II regions. The residue that corresponds to Ile156 in H-Twist is Ile550, shown in red. The residue sits at a key position of the interface, forming bonds with seven residues in Max, shown in green.

The basic helix-loop-helix domain of H-Twist shares 45% sequence identity with the c-Myc transcription factor that was crystalized by Nair *et al. *[49] (Figure 6). The structure shows a dimer of c-Myc and Max bound to DNA. The c-Myc/Max dimerization is essential for the transcriptional regulation. The Ile156Val mutation is located at the core of the interaction interface. Although the Ile156Val mutation constitutes a biochemically similar substitution, reflected by the relatively high frequency of valine at this position in other helix-loop-helix proteins (conservation scores 2.76 for isoleucine and 1.23 for valine), the change in volume could slightly change the interaction propensity. Correspondingly, the Ile156Val mutation causes a mild form of Baller-Gerold syndrome.

## Conclusion

Protein interactions can be the root cause of genetic pathologies, yet their significance for health and disease remain to be quantified. In this first comprehensive survey, we identified both known and putative mutations that affect protein interactions.

We devised an automated method to predict interaction related residues in proteins. It uses sequence-based homology detection to correlate mutations to structures of interacting proteins. When applied to disease causing mutations from OMIM and UniProt, our algorithm yields a set of 1,428 interaction-related mutations. This suggests that approximately 4% of mutations could have an effect on protein interactions. In comparison to non-interaction related mutations, we observed an enrichment for dominant or co-dominant mutations in both the curated as well as in the predicted set. Furthermore, there appear to be subtle differences in the residue composition between interaction related mutations and disease related mutations in general.

Our curated list of interaction-related diseases underlines that a wide variety of proteins are susceptible to mutations that alter protein interaction. The list provides examples to categorize mutations according to their functional and molecular properties. We found that numerous LOF mutations feature dominant inheritance, suggesting that stoichiometric imbalances or failing collaborative mechanisms in protein complexes frequently result in a dominant phenotype.

Further mutagenesis and protein interaction experiments on selected examples from our predicted set could shed new light on the molecular mechanisms behind human genetic diseases. In turn, knowledge of more cases of interaction-related disease will help to improve the accuracy of prediction algorithms.

## Materials and methods

### *i*Pfam

*i*Pfam [16] is a database of interactions between Pfam families. It is derived by identifying Pfam families on protein structures in the PDB. All cases of residue-to-residue proximity between two family instances of less than 6 Å distance are collected. *i*Pfam version 20 was employed, containing 3,020 interacting domain pairs composed of 2,147 individual domains.

### Homology detection and alignment

Protein sequences were screened for *i*Pfam families using hidden Markov models with the pfam_scan.pl script [50]. For each identified family, matching regions were aligned to structures in which the respective *i*Pfam family had been found to interact using hmmalign from the HMMER package [51]. The percentage sequence identity between all pairs of aligned regions was calculated using the exact implementation in the Bio::SimpleAlign BioPerl module.

### Conservation

Residue conservation was extracted directly from the Pfam HMM that matched a sequence region. Using hmmfetch from the HMMER package, we mapped columns in the alignment back to states in the profile hidden Markov model. The HMM Perl library [52] was employed to extract the emission profiles and background probabilities. For every mutation, the log-odds score of the original and the mutated residue were reported.

### Alanine scanning database

The ASEdb database [21] contains 101 experiments extracted from 74 publications that are available online [53]. There were 3,010 residue mutations recorded. Mutations leading to incorrectly folded proteins or premature degradation were excluded from ASEdb if this information was available in the source publication. In order to use hidden Markov models to search for *i*Pfam domains, protein sequences corresponding to the gene name annotated in ASEdb were retrieved from UniProt. Only proteins for which all amino acid annotations in ASEdb matched the sequence were included. For 1,202 residue mutations, a UniProt sequence could be identified. There were 439 mutations from experiments that involved an antibody as the binding partner and were subsequently removed. An additional 81 mutations extracted from recent publications were added manually.

### Disease mutations

Mutation data were collected from UniProt [54] and OMIM [6]. For UniProt, human sequences with variation information were acquired using SRS [55]. The analysis was restricted to disease-related single residue mutations by regular expression matching on the variant description line in UniProt entries. OMIM (omim.txt.Z, genemap) and Entrez gene mappings (mim2gene, gene2refseq.gz) were downloaded from the NCBI FTP server [56] as flat files. Mapping OMIM entries to a reference sequence is not trivial. To accomplish this, protein sequences for every gene ID reference in the OMIM entry were acquired from NCBI and UniProt through SRS. To identify the correct co-ordinate system that fits an OMIM entry, combinations of signal peptide and other post-translationally cleaved regions were considered. If the amino acid annotations in the OMIM entries for a gene matched the residues at the respective position in the reference sequence, that co-ordinate system was used.

### Compiling the curated set of interaction-related mutations

In order to identify known interaction-related mutations, all OMIM 'Description' fields were searched for keywords such as 'interaction', 'binding' or 'complex'. For all matching mutations, the available literature was manually evaluated. Subsequently, PubMed was searched for the same keywords. Lastly, cases that were identified by the prediction method were added if they were found to be known in the literature. If a mutation was shown to be causative and described to directly affect a protein interaction, it was added to the list. Mutations that lead to folding errors were excluded from the data set.

### Graphics

Three-dimensional protein images were prepared using VMD [57] and rendered with PovRay [58].

## Abbreviations

Δ*G*, Gibbs free energy; GOF, gain of function; LOF, loss of function; nsSNP, non-synonymous single nucleotide polymorphism; OMIM, Online Mendelian Inheritance in Man; PDB, Protein Data Bank.

## Authors' contributions

BSB wrote all software and carried out all the analyses. AB contributed to the design and interpretation of the study. Both authors wrote and approved the manuscript.

## Additional data files

The following additional data are available. Additional data file 1 is an Excel spreadsheet listing all 1,428 predicted interacting mutations and the corresponding structural templates, homologous interactions and surface accessibilities. Additional data file 2 is an Excel spreadsheet containing 119 disease mutations linked to protein interaction defects, derived from the scientific literature. Additional data file 3 contains a figure showing the distributions of residue frequencies for all mutations in OMIM and Uniprot (wild type), the predicted set (wild type), the curated set, for interface residues as described by Chakrabarti *et al. *[38], the whole of UniProt and for residues from ASEdb with ΔΔ*G *> 2.

## Supplementary Material

Additional data file 1 All 1,428 predicted interacting mutations and the corresponding structural templates, homologous interactions and surface accessibilities. Click here for file

Additional data file 2 119 disease mutations linked to protein interaction defects, derived from the scientific literature. Click here for file

Additional data file 3 Error bars for the predicted set were calculated by randomly resampling 1,428 residues from all mutations 1,000 times and calculating the standard deviation. Click here for file
