# Supplementary figures and images for: Protein interactions in human genetic diseases
Source: Genome Biol. 2008 Jan 16;9(1):R9. doi: 10.1186/gb-2008-9-1-r9 (PMC2395246; doi:10.1186/gb-2008-9-1-r9)

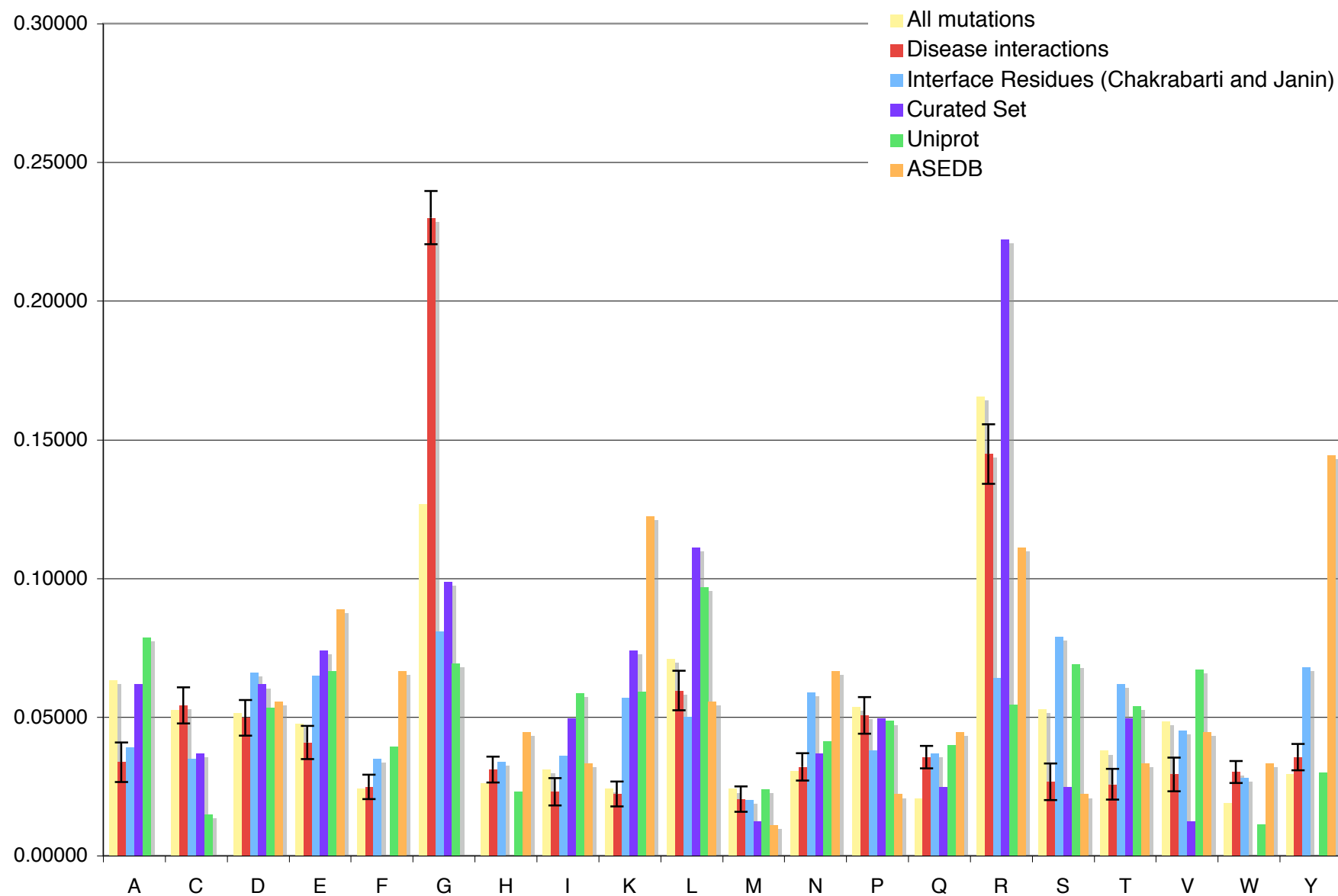

Supplement: Additional data file 3 — Error bars for the predicted set were calculated by randomly resampling 1,428 residues from all mutations 1,000 times and calculating the standard deviation. [file gb-2008-9-1-r9-S3.pdf]
